# Dr. John Berrien Lindsley

**Published:** 1898-02

**Authors:** 


Dr. John Berrien Lindsley, of Nashville, died at his home in that city, December 7, 1897, aged 75 years. Dr. Lindsley was a prominent sanitarian, having been secretary of the Tennessee board of health for many years. He was a member of several scientific societies at home and abroad.

